# Supplementary material for: MetaRibo-Seq measures translation in microbiomes
Source: Nat Commun. 2020 Jun 29;11:3268. doi: 10.1038/s41467-020-17081-z (PMC7324362; doi:10.1038/s41467-020-17081-z)
Supplement: Supplementary file 10 — Supplementary Data 7 [file 41467_2020_17081_MOESM10_ESM.zip › File2/Confidence_VeryHigh_Taxonomy/66785_out.krona.html]

Javascript must be enabled to view this page.

members
magnitude
magnitudeUnassigned
count
unassigned
taxon
rank

66785\_out

5

5
superkingdom
2

phylum
4
1239

186801
class
4

186802
order
4

186803
2
family

2
species

SRS050941\_contig\_number\_5494SRS142923\_contig\_number\_8834
39491

186806
family
2

1730
2
genus

species
2
142586

SRS050998\_contig\_number\_15284SRS144362\_contig\_number\_contig-100\_419.244420

1
phylum
201174

1
class
1760

order
1
85006

85023
1
family

1
genus
46352

399736

SRS023346\_contig\_number\_contig-100\_6852.6852
1
species
